# Parent Satisfaction with Family-Based Addiction Prevention and Psychosocial Health Promotion Services: A Cross-Sectional Survey at a Community Prevention Center in Greece

**DOI:** 10.3390/healthcare14152278

**Published:** 2026-07-26

**Authors:** Ioanna Zormpa, Constantinos Togas, John Fanourgiakis, Christos Ntais

**Affiliations:** 1 Healthcare Management Program, School of Social Sciences, Hellenic Open University, 26335 Patras, Greece; 2 School of Science and Technology, Hellenic Open University, 26335 Patras, Greece; togkas.konstantinos@ac.eap.gr; 3 Department of Management Science and Technology, Hellenic Mediterranean University, 72100 Agios Nikolaos, Greece; jfanourgiakis@hmu.gr; 4 Healthcare Management Program, School of Economics & Management, Open University of Cyprus, Nicosia 2220, Cyprus

**Keywords:** addiction prevention, parent training, psychosocial health promotion, satisfaction survey, parent satisfaction, CSQ-8, community prevention

## Abstract

**Background/Objectives:** Satisfaction with health and psychosocial services is a key indicator of perceived quality and acceptability, but it does not show whether services improve parenting, family functioning or youth outcomes. It is rarely assessed systematically in community-based addiction prevention and psychosocial health promotion services. This primarily descriptive cross-sectional survey assessed parent-reported satisfaction with family-based addiction prevention and psychosocial health promotion services delivered by a Greek community prevention center. **Methods:** An anonymous cross-sectional online survey was completed by 110 of 250 invited parents (44%) who had participated in family-based programs delivered by the Prevention Center of the Regional Unit of Achaia (“Kallipolis”) during the 3 years immediately preceding data collection. Satisfaction was measured with a licensed Greek-language version of the Client Satisfaction Questionnaire (CSQ-8). The primary analyses were descriptive, and sample-specific internal consistency was assessed. Because only six respondents had scores below the upper descriptive range, a two-predictor Firth penalized logistic regression was fitted solely for exploratory, hypothesis-generating purposes. **Results:** The mean CSQ-8 score was 29.35 (SD = 2.84), the median was 30 (interquartile range = 28–31.75), and 104 respondents (94.5%) scored 25–32. Internal consistency was good (Cronbach’s alpha = 0.82), although the distribution showed a pronounced ceiling effect. The exploratory model suggested an association between complete attendance and an upper-range score; however, the reference attendance group contained only three participants, producing an extremely imprecise estimate and precluding reliable interpretation of the magnitude of the association. **Conclusions:** Respondents reported very high perceived satisfaction and acceptability. The findings are primarily descriptive, may be affected by nonresponse, selection, and recall bias, and do not demonstrate an improvement in parenting, family functioning or youth outcomes. Satisfaction monitoring should be combined with measures of reach, retention, implementation fidelity, and participant, family and youth outcomes.

## 1. Introduction

Science-based prevention is a core component of public health responses to substance use and related psychosocial risks. International standards emphasize that evidence-informed prevention extends beyond information provision and aims to strengthen protective factors, reduce risk factors and support healthy development across family, school and community contexts [1]. In Europe, prevention science has also been promoted through standardized training and quality-improvement initiatives intended to strengthen the professional prevention workforce and improve the selection and implementation of evidence-informed interventions [2].

Family and parenting interventions are central to this prevention approach. Parenting skills, positive family relationships, consistent rules, emotional support and parental monitoring are protective factors that may delay or reduce young people’s substance use and other risk behaviors. Systematic reviews suggest that parent- and family-based interventions can improve parenting and youth outcomes, particularly when programs go beyond knowledge transfer and include skills development, active learning and sufficient program intensity [3,4,5,6,7,8]. In this context, program intensity refers to the planned intervention dose or exposure, including the number, frequency and duration of sessions and the opportunities provided for active skills practice. For community organizations that provide such programs, assessing parents’ experiences is important both for quality assurance and for improving engagement and retention.

Satisfaction with services is widely used as an indicator of perceived quality in health, mental health and social care. Donabedian’s model conceptualizes quality through three linked domains: structure, referring to the organizational conditions, workforce and resources that support care; process, referring to what is delivered and how it is delivered; and outcome, referring to changes in health, functioning or service-user perceptions [9,10]. Satisfaction can contribute to process and outcome assessment, but it does not by itself establish technical quality or demonstrate changes in behavioral or health outcomes [11,12,13,14]. It nevertheless provides useful information about acceptability, perceived usefulness, access, communication and the extent to which services meet users’ needs. In prevention programs, satisfaction data can help organizations identify barriers to participation, adapt services to local populations and monitor whether services remain responsive over time.

The measurement of parental perspectives in parenting programs remains less developed than the measurement of program outcomes. A scoping review by Bjorknes and Ortiz-Barreda found that parents’ voices are not consistently incorporated into studies of parenting programs, despite high satisfaction being reported in studies that do measure it [15]. Structured satisfaction monitoring should therefore be considered alongside indicators of reach, implementation and outcomes, rather than treating satisfaction as a stand-alone endpoint.

In Greece, Prevention Centers for Addictions and Promotion of Psychosocial Health constitute a nationwide network of community-based organizations operating in collaboration with the National Organization for the Prevention and Management of Addictions (ΕOPAΕ), formerly known as the Organisation Against Drugs (OΚAΝA), as well as local authorities. The network includes 75 prevention centers covering the country’s 13 regions, and their services include universal, selective and indicated prevention activities in schools, families and communities. According to the 2023 annual report of the National Center for Documentation and Information on Drugs (EKTEPN), prevention centers reach thousands of children, adolescents, parents, educators and community members each year [16]. However, systematic and comparable measurement of service-user satisfaction remains limited.

The Prevention Center of the Regional Unit of Achaia “Kallipolis” is based in Patras and provides prevention and psychosocial health promotion programs across the Regional Unit of Achaia. Its family-based services include seminars and groups for parents of preschool and school-age children, parents of adolescents, single parents and couples. Programs are differentiated according to child developmental stage and family context. The services are specified within the center’s Five-Year Scientific Program 2021–2025 and are informed by prevention-science principles that emphasize strengthening family protective factors, interactive skills development and attention to developmental and community context [1,2,5,17]. They use experiential adult-learning methods, facilitated group processes and skills practice to support communication, emotional awareness, boundary setting, self-esteem and parents’ role in prevention.

The primary objective of this cross-sectional satisfaction survey was to assess satisfaction among parents who had received family-based services from “Kallipolis”. A secondary, explicitly exploratory objective was to examine whether the satisfaction pattern was associated with selected service-use factors. By focusing on a community prevention setting, the study contributes to the limited international literature on parent-reported satisfaction in addiction prevention and psychosocial health promotion services.

## 2. Materials and Methods

### 2.1. Study Design and Reporting Approach

This was a cross-sectional survey of parents who had participated in family-based prevention programs delivered by “Kallipolis”. Where applicable, this manuscript was structured according to the Strengthening the Reporting of Observational Studies in Epidemiology (STROBE) recommendations for cross-sectional studies [18].

### 2.2. Setting

“Kallipolis” is one of the community Prevention Centers for Addictions and Promotion of Psychosocial Health operating in Greece. It provides free services to children, adolescents, parents, educators, young adults, volunteers and other community groups. Family programs are organized for specific developmental stages or family contexts. The family-based programs included in this survey were delivered mainly in Patras, where the center is based, and in other urban or semi-urban areas of Achaia. Program venues included the center’s offices, school facilities and spaces provided by municipalities or collaborating organizations.

### 2.3. Programs Included in the Survey

The programs included in the survey were group-based seminars for parents of preschool and school-age children, parents of adolescents, single parents and couples. Their planned intensity generally comprised 6–8 weekly meetings of approximately 2.5 h each, with groups of approximately 20–25 participants. The content was developed within “Kallipolis” Five-Year Scientific Program 2021–2025 [17]. The services did not constitute one branded, uniformly manualized intervention; rather, they drew on international family-based prevention guidance and on the prevention-science emphasis on modifiable family risk and protective factors, active skills learning and sufficient exposure to program content [1,2,5]. Content addressed the developmental needs of children and parents, effective communication and active listening, empathy, boundary setting, emotion recognition and management, self-esteem, family relationships, couple dynamics and the role of parents in preventing addictive behaviors. The delivery approach combined experiential adult education, facilitated group discussion, role play, creative and reflective activities and brief theoretical input. The study did not collect a process measure distinguishing self-selection, referral-led entry or staff-guided program selection.

### 2.4. Participants and Recruitment

Eligible participants were parents who had participated in at least one “Kallipolis” family-based program during the 3 years immediately preceding the January–February 2025 survey; this eligibility window referred to the 3 years before data collection rather than to calendar years or program cycles. All 250 parents in the eligible pool were invited by email to complete an anonymous online questionnaire created in Google Forms. A total of 110 parents completed the questionnaire, corresponding to a response rate of 44.0%. Participation was voluntary, and no identifying information was collected. The survey did not record the exact date or year of program participation, and individual-level information on nonrespondents was unavailable. Consequently, satisfaction could not be compared by time since participation or between respondents and nonrespondents.

### 2.5. Measures

The questionnaire consisted of three sections. The first section collected sociodemographic characteristics: sex/gender, age, place of residence, marital/family status, number of children, employment status, educational attainment and annual personal income. The second section collected information on service use, including type of program attended, number of meetings attended, previous use of “Kallipolis” services, total duration of service use, service location, number of staff members involved, participation of other family members and source of information about the center. Program type was recorded in mutually exclusive categories: four specific single-program categories and two generic combination categories (“two programs” and “more than two programs”). The component programs within the combination categories were not recorded. The third section included a licensed Greek-language version of the Client Satisfaction Questionnaire (CSQ-8), a brief eight-item service-satisfaction instrument developed for health and human services and used across service settings [19,20,21,22]. We did not identify a published formal validation study for this specific Greek version and therefore sample-specific internal consistency was assessed. The full study questionnaire, including the CSQ-8 text and response options, is reproduced in the Supplementary Materials with the permission held for this study and the required copyright and permission notice.

The CSQ-8 contains eight items scored on four-point response scales and yields a total score ranging from 8 to 32, with higher scores indicating greater satisfaction. The total score on its original 8–32 scale was the primary satisfaction summary. For descriptive presentation only, scores were also divided into three equal-width bands: lower (8–16), middle (17–24) and upper (25–32). These bands were not treated as validated clinical cutoffs.

### 2.6. Ethical Considerations

The study was conducted in the context of the M.Sc. program “Health Care Management” of the Hellenic Open University. The study protocol was approved by the competent body of the program (protocol number 161856/23-10-2024). The online questionnaire included participant information, and a written informed consent statement was obtained from all participants. Respondents could proceed only after indicating consent. Data were collected anonymously and used exclusively for research purposes.

### 2.7. Statistical Analysis

Responses were exported from Google Forms to Microsoft Excel, checked for completeness, coded and analyzed using IBM SPSS Statistics version 24 and R version 4.3. The primary analyses were descriptive. Categorical variables are presented as frequencies and percentages. Age and CSQ-8 total scores are summarized using means, standard deviations, medians, interquartile ranges and observed ranges where appropriate. Internal consistency of the eight CSQ-8 items in this sample was assessed using Cronbach’s alpha. To retain information from the original 8–32 scale, CSQ-8 medians and interquartile ranges were also examined across meeting-attendance and previous-service-use categories. For the exploratory regression only, the score was dichotomized as upper range (25–32) versus lower or middle range (≤24). This threshold-dependent dichotomization discards within-band information and intensified the sparse-outcome problem; the resulting model was therefore not used for confirmatory inference. Only six participants were outside the upper range, which was insufficient to support a fully adjusted multivariable model including sociodemographic variables and program type. The regression was deliberately restricted to a two-predictor Firth penalized logistic regression as a bias-reduction approach for sparse binary data [23]. Meeting attendance and previous use of “Kallipolis” services were selected a priori as service-utilization characteristics proximal to engagement and service experience. The model adjusted only for these two predictors. Importantly, the reference attendance category contained three participants, of whom one was in the upper score range, and two were not. Firth penalization reduces small-sample bias and can accommodate separation but cannot compensate for the very limited information in this group; estimates are therefore highly sensitive to individual observations. Adjusted odds ratios (aORs), profile-likelihood 95% confidence intervals (CIs), and penalized likelihood-ratio *p* values are reported solely as exploratory, hypothesis-generating results.

## 3. Results

### 3.1. Participant Characteristics

The final sample included 110 parents. Most respondents were women (83.6%), and the mean age was 47.04 years (SD = 7.05; range: 34–66 years). The sample had a high educational profile: 44.5% had a tertiary education, and 36.4% had postgraduate or doctoral education. Most participants were married or in a civil partnership (70.0%), had two children (58.2%), lived in an urban area (70.0%) and reported an annual individual income of EUR 10,001–20,000 (51.8%). Full sociodemographic characteristics are presented in Table 1.

### 3.2. Services Received

The most frequent specific single-program category was the seminar/group for parents of preschool and school-age children (43/110, 39.1%), whereas eight respondents (7.3%) reported the parent group for adolescents as their only program. However, 48 respondents (43.6%) were recorded in generic multi-program categories whose component programs were not specified. Consequently, the single-program percentages describe mutually exclusive survey categories and should not be interpreted as total exposure to each program or as center-wide utilization rates. Attendance was high: 83.6% reported attending all meetings, and 13.6% attended more than half of the meetings. Most respondents received services for 1–3 months (65.5%), in Patras (75.5%), and at the center’s offices (81.8%). Half reported that another family member had also received services from the center. Service characteristics are presented in Table 2.

### 3.3. Satisfaction Scores

The distribution of CSQ-8 scores showed a pronounced ceiling effect. The mean score was 29.35 (SD = 2.84; median = 30.0; interquartile range = 28.0–31.75; observed range = 19–32). Internal consistency in this sample was good (Cronbach’s alpha = 0.82). In the descriptive equal-width bands, 104 participants (94.5%) were in the upper range (25–32), six (5.5%) were in the middle range (17–24), and none were in the lower range (8–16) (Table 3). These bands are descriptive and are not validated clinical thresholds.

### 3.4. Exploratory Associations with Satisfaction

To retain the total-score information, satisfaction was first summarized within the service-use categories. The three participants who attended fewer than half of the meetings had CSQ-8 scores of 19, 19 and 30. The median was 30 (interquartile range = 25.5–31) among the 15 participants who attended more than half of the meetings and 30.5 (interquartile range = 28–32) among the 92 who attended all meetings. The median was 30 (interquartile range = 27–31) among participants without previous “Kallipolis” service use and 31 (interquartile range = 30–32) among those with previous use. For the dichotomized exploratory analysis, one of three participants who attended fewer than half of the meetings was in the upper score range, compared with 13 of 15 who attended more than half and 90 of 92 who attended all meetings. Within the two-predictor Firth model, complete attendance was associated with an upper-range score relative to the three-person reference group (aOR = 58.53, 95% CI: 5.71–953.90, *p* = 0.001) (Table 4). The confidence interval spans more than two orders of magnitude and indicates extreme imprecision rather than a precise estimate of the association’s magnitude. Because each observation represented one-third of the reference group, the point estimate and interval were highly sensitive to individual outcomes. The estimate for attendance at more than half of the meetings was also imprecise (aOR = 9.14, 95% CI: 0.84–155.54, *p* = 0.069). Previous service use was not clearly associated with the dichotomized outcome after adjustment for attendance (aOR = 2.46, 95% CI: 0.39–30.40, *p* = 0.355).

## 4. Discussion

This primarily descriptive cross-sectional survey examined parent-reported satisfaction with family-based addiction prevention and psychosocial health promotion services provided by a Greek community prevention center. Respondents reported scores close to the upper end of the CSQ-8, and 94.5% were in the upper descriptive score range. Cronbach’s alpha of 0.82 supported good internal consistency in this dataset. At the same time, the pronounced ceiling effect limited discrimination between participants. The findings indicate high perceived acceptability and usefulness among those who responded; they should not be interpreted as an objective measure of service quality or as evidence that the programs improved parenting, family functioning or youth outcomes.

The high satisfaction scores are consistent with international evidence on parenting programs. In their scoping review, Bjorknes and Ortiz-Barreda reported that parents generally express high satisfaction when this outcome is measured, although parent perspectives are not routinely incorporated into studies of parenting programs [15]. The present findings add descriptive evidence that respondents perceived the family-based services as acceptable and responsive to their needs. However, the high scores may also reflect ceiling effects and selective participation by parents with more favorable experiences.

In the exploratory two-predictor model, complete attendance was associated with an upper-range score after adjustment only for previous service use. This finding should not be interpreted as an independent or causal association because sociodemographic characteristics, program type, motivation, perceived need and program fit were not included. Moreover, the comparison relied on a reference group of only three participants, with one upper-range and two lower/middle-range outcomes. Although Firth penalization reduced small-sample bias and addressed potential separation, it could not create information that was absent from this group. The very wide 95% confidence interval (5.71–953.90), therefore, represents extreme uncertainty, and the point estimate should not be interpreted as a reliable estimate of the magnitude of the attendance–satisfaction association. Reverse causation is also plausible: positive experiences may promote continued attendance, rather than attendance alone producing satisfaction.

Participants with previous “Kallipolis” service use had a slightly higher median total score than those without previous use, but the dichotomized Firth estimate was highly imprecise and did not provide clear evidence of an association after adjustment for attendance. Prior users may differ in trust, familiarity, motivation or service needs; consequently, previous participation is best viewed as a contextual characteristic rather than as an independent predictor in this dataset.

The sample profile provides useful information for service planning. Women represented 83.6% of respondents, leaving fathers as a small proportion of the respondent sample. Because the sex/gender distribution of the full eligible service-user population was unavailable, this finding cannot establish lower reach among fathers. Nevertheless, the low representation of fathers among respondents is consistent with a broader evidence base in which parenting interventions have predominantly engaged mothers and identifies father participation as a priority for future recruitment and satisfaction monitoring [7]. Most respondents also had tertiary or postgraduate education. Future recruitment and satisfaction-monitoring strategies should therefore consider practical and cultural barriers, language and health-literacy demands, program timing and recruitment channels that may affect participation by fathers and parents with lower educational attainment [24]. A further noteworthy pattern was the difference between the preschool/school-age single-program category (43/110, 39.1%) and the adolescent–parent single-program category (8/110, 7.3%). This warrants attention because adolescence is a developmental period in which substance use experimentation and other risk behaviors become particularly salient [25,26]. Nevertheless, the difference cannot be interpreted as evidence that adolescent–parent programs were less used across the center: 48 respondents (43.6%) were in generic multi-program categories that did not identify their component programs, and the study did not include administrative denominators such as the number of programs offered, inquiries, enrolments or completions by program and year. The observed difference should therefore be treated as a service-planning signal rather than a center-wide utilization rate.

The findings have practical implications at both the national and international levels. For Greek prevention centers, routine use of a common satisfaction measure could support quality improvement if response rates, administration timing and score distributions are reported transparently. Feedback procedures should actively include program completers and non-completers, offer anonymous and accessible response modes and monitor the representation of fathers, parents with lower educational attainment and residents outside major urban areas. Administrative monitoring should also disaggregate the number of program offerings, capacity, inquiries, enrolments, attendance and completion by year and child developmental stage. If such monitoring confirms lower reach among parents of adolescents, centers could test targeted communication, stronger referral partnerships and flexible delivery arrangements. Satisfaction results should be integrated into a broader quality-monitoring framework that includes reach, attendance and retention, implementation fidelity, staff training, pre-post participant outcomes and follow-up indicators of family functioning and youth risk and protective factors. Internationally, community prevention services would benefit from harmonized core monitoring domains and comparable reporting of satisfaction, implementation and outcomes. Such an approach would permit cautious benchmarking and shared learning without treating favorable satisfaction ratings as evidence of improvement in participant, family or youth outcomes [1,2].

Several methodological issues qualify for interpretation. Satisfaction studies often produce high positive ratings because of gratitude, low expectations, reluctance to criticize, social desirability and selective response among people with favorable experiences [11,12,13,14]. Here, only 110 of 250 invited parents responded, and no characteristics of nonrespondents were available. Nonresponse or selection bias may therefore have contributed to the high observed scores, particularly if parents who discontinued programs or had less favorable experiences were less likely to respond; the direction and magnitude of this bias cannot be quantified from the available data. The 3-year retrospective eligibility window may also have introduced recall variation. Because the survey did not record the date of participation, responses could not be examined according to the time elapsed since the program. Program type was captured with generic multi-program categories, and center-wide administrative denominators were not analyzed; therefore, the observed program-category distribution cannot establish relative demand or use. Finally, dichotomization of the ceiling-skewed score left only six non-upper outcomes and a three-person attendance reference group. The Firth model should therefore be viewed as exploratory and hypothesis-generating, not as a stable multivariable estimate. The cross-sectional design additionally precludes causal interpretation and leaves substantial potential for reverse causation and unmeasured confounding.

Future research should extend this work across multiple prevention centers and geographical regions, include larger and more diverse samples and combine quantitative satisfaction measures with qualitative feedback. Future satisfaction studies should record program dates and administer questionnaires at standardized intervals so that recent and earlier participants can be compared. They should record the specific component programs attended by multi-program users and link survey data, where ethically permissible, with annual administrative indicators of program offerings, capacity, inquiries, enrolment, attendance and completion by child developmental stage. They should also make specific efforts to include non-completers, fathers and parents with lower educational attainment. Most importantly, satisfaction should be examined alongside implementation indicators, including engagement and retention, and objective outcomes, such as knowledge or skill acquisition, family-level outcomes and longer-term youth risk and protective factors.

## 5. Strengths and Limitations

A strength of this study is its focus on a real-world community prevention service, a setting in which systematic satisfaction and quality monitoring are often underreported. The study used a standardized and widely recognized satisfaction instrument, reported sample-specific internal consistency and described both sociodemographic and service-use characteristics.

The limitations should also be acknowledged. First, this was a single-center study, and findings may not generalize to all prevention centers or countries. Second, the response rate was 44.0%, no information was available for nonrespondents, and selection or nonresponse bias may have inflated satisfaction if parents who discontinued programs or had less favorable experiences were underrepresented. Third, satisfaction was measured retrospectively and by self-report across a 3-year eligibility window. Because the exact participation date was not collected, recall effects and variation by time since participation could not be assessed. Fourth, the CSQ-8 distribution had a pronounced ceiling effect. Dividing the total score into bands lost information and produced only six non-upper outcomes. The attendance reference group contained only three participants, so even the Firth estimates were highly unstable and sensitive to individual observations. Fifth, the exploratory model adjusted only for attendance and previous service use; it did not control for sociodemographic characteristics, program type, motivation or other potential confounders. Sixth, the sample was predominantly female and highly educated, limiting conclusions about fathers and parents with lower educational attainment. Seventh, the generic multi-program categories did not identify their component programs, and data on center-wide records of program offerings, capacity, inquiries, enrolments and completions were not collected; relative program use, including the reach of adolescent–parent programs, could not be estimated. Finally, satisfaction reflects perceived quality and acceptability and should not be used as a proxy for improvements in parenting, family functioning or youth risk and protective factors.

## 6. Conclusions

Parents who responded to this cross-sectional satisfaction survey reported CSQ-8 scores near the upper end of the scale, indicating high perceived satisfaction and acceptability among respondents. The exploratory Firth model generated a hypothesis that complete attendance may be related to an upper-range score, but the six non-upper outcomes and the three-person reference group prevented a precise, fully adjusted or causal estimate. The study therefore supports the value of systematic satisfaction monitoring while also showing why satisfaction must be interpreted alongside response patterns, reach, retention, implementation fidelity, and participant, family and youth outcomes. For policy and practice, routine monitoring systems should seek feedback from both completers and non-completers, use targeted strategies to increase participation by fathers and parents with lower educational attainment, and monitor program reach by child developmental stage, particularly whether parents of adolescents are being adequately reached. Larger multi-center studies with standardized assessment timing and integrated satisfaction, implementation and outcome indicators are needed.

## Figures and Tables

**Table 1:** Sociodemographic characteristics of participants (N = 110).

Characteristic	Category/Statistic	n (%) or Value
Gender	Men	18 (16.4)
	Women	92 (83.6)
Age	Mean (SD)	47.04 (7.05)
	Minimum-maximum	34–66
Education	Secondary education	21 (19.1)
	Tertiary education	49 (44.5)
	Postgraduate/doctoral degree	40 (36.4)
Employment status	Public-sector employee	51 (46.4)
	Private-sector employee	23 (20.9)
	Self-employed	19 (17.3)
	Retired	7 (6.4)
	Unemployed	10 (9.1)
Family status	Single	7 (6.4)
	Married/civil partnership	77 (70.0)
	Divorced	21 (19.1)
	Widowed	5 (4.5)
Number of children	1	33 (30.0)
	2	64 (58.2)
	3	12 (10.9)
	4	1 (0.9)
Place of residence	Urban	77 (70.0)
	Semi-urban	23 (20.9)
	Rural	10 (9.1)
Annual individual income (EUR)	0–10,000	21 (19.1)
	10,001–20,000	57 (51.8)
	20,001–30,000	26 (23.6)
	>30,001	6 (5.5)

**Table 2:** Program and service-use characteristics (N = 110).

Service Variable	Category	n (%)
Program(s) attended	Parent group for preschool/school-age children	43 (39.1)
	Parent group for adolescents	8 (7.3)
	Group for single parents	9 (8.2)
	Couples group	2 (1.8)
	Two “Kallipolis” programs	36 (32.7)
	More than two “Kallipolis” programs	12 (10.9)
Attendance	Less than half of meetings	3 (2.7)
	More than half of meetings	15 (13.6)
	All meetings	92 (83.6)
Previous “Kallipolis” services	Yes	41 (37.3)
	No	69 (62.7)
Total duration of services	1–3 months	72 (65.5)
	3–6 months	22 (20.0)
	6–12 months	11 (10.0)
	>12 months	5 (4.5)
Place of service delivery	Patras	83 (75.5)
	Other location	27 (24.5)
Venue	Prevention center offices	90 (81.8)
	Other venue	20 (18.2)
Number of staff members involved	One staff member	41 (37.3)
	More than one staff member	69 (62.7)
Other family member received services	Yes	55 (50.0)
	No	55 (50.0)
Information source	Social media	13 (11.8)
	Friends/acquaintances	31 (28.2)
	Other single source	9 (8.2)
	Combination of sources	57 (51.8)

**Table 3:** Descriptive CSQ-8 score bands (N = 110).

CSQ-8 Score Band	Score Range	n (%)
Lower score range	8–16	0 (0.0)
Middle score range	17–24	6 (5.5)
Upper score range	25–32	104 (94.5)

**Table 4:** Exploratory, hypothesis-generating Firth penalized logistic regression for an upper-range CSQ-8 score (25–32).

Predictor	Comparison/Coding	aOR	95% CI	*p* Value
Meeting attendance	More than half (n = 15) vs. less than half (n = 3)	9.14	0.84–155.54	0.069
Meeting attendance	All meetings (n = 92) vs. less than half (n = 3)	58.53	5.71–953.90	0.001
Previous “Kallipolis” services	Yes (n = 41) vs. no (n = 69)	2.46	0.39–30.40	0.355

Note: The outcome was an upper-range CSQ-8 score (25–32) versus a lower or middle score (≤24). These equal-width bands are descriptive and are not validated clinical cutoffs. aOR = adjusted odds ratio; CI = confidence interval. Odds ratios are adjusted only for the predictors shown in the table; sociodemographic variables and program type were not included. Confidence intervals are profile-likelihood CIs, and *p* values are penalized likelihood-ratio *p* values. The attendance reference group contained only three participants (one upper-range and two lower/middle-range outcomes). Firth penalization reduces small-sample bias and can accommodate separation but does not remove the substantial instability caused by this very small reference group; the estimates and their magnitudes should therefore be interpreted as exploratory and highly imprecise.

## Data Availability

The data are not publicly available because public repository deposition was not specified in the participant information sheet. Data may be made available by the corresponding author on reasonable request, subject to ethical and legal restrictions.

## Supplementary Materials

The following supporting information can be downloaded at: https://www.mdpi.com/article/10.3390/healthcare14152278/s1, the full study questionnaire, including the CSQ-8 text and response options, reproduced under the permission held for this study and with the required copyright and permission notice.
