# Supplementary material for: Parent Satisfaction with Family-Based Addiction Prevention and Psychosocial Health Promotion Services: A Cross-Sectional Survey at a Community Prevention Center in Greece
Source: Healthcare (Basel). 2026 Jul 26;14(15):2278. doi: 10.3390/healthcare14152278 (PMC13466087; doi:10.3390/healthcare14152278)
Supplement: Supplementary file 1 [file healthcare-14-02278-s001.zip › healthcare-4384657-supplementary.pdf]

Supplementary Material S1

Study Questionnaire

Section S1. Demographic Characteristics

Question	Response options
1. Sex/gender	Woman; Man; Other
2. Age	Numeric response
3. Place of residence	Rural; Semi-urban; Urban
4. Family status	Single; Divorced; Married/civil partnership; Widowed; Other
5. Number of children	1; 2; 3; 4; Other
6. Employment status	Unemployed; Public-sector employee; Private-sector employee; Self-employed; Retired; Other
7. Educational level	Primary school graduate/Lower secondary school graduate/Upper secondary school graduate; University/technological institute graduate; Postgraduate/doctoral degree holder; Other
8. Annual individual income (EUR)	0-10,000; 10,001-20,000; 20,001-30,000; More than 30,000

Section S2. Services Received from Kallipolis Prevention Center

**Question**	**Response options**
9. Please indicate the type of services you have received from Kallipolis Prevention Center.	Participation in a seminar/group for parents of preschool and school-age children; Participation in a seminar/group for parents of adolescents; Participation in a seminar/group for single parents; Participation in a seminar/group for couples; Combination of 2; Combination of >2; Other
10. How many meetings of the seminar/group did you attend?	Less than half of the meetings; More than half of the meetings; All meetings; Other
11. Had you previously received any other type of service from Kallipolis Prevention Center?	No; Yes
12. Please indicate the total duration of the services you have received from Kallipolis Prevention Center.	1-3 months; 3-6 months; 6-12 months; >12 months; Other
13. Please indicate the place where you received the services.	Non-Patras; Patras
14. Please indicate the venue where you received the services.	Non-Prevention Center offices; Prevention Center offices
15. From how many Kallipolis Prevention Center staff members have you received services?	One staff member; >1 staff members; Other
16. Apart from you, has another member of your family received services from Kallipolis Prevention Center?	No; Yes; If yes, please indicate which family member(s) received services (Children; Partner/Spouse/Ex; Children + Partner)
17. How did you learn about Kallipolis Prevention Center?	Social media; Acquaintances/relatives/friends; Another department/agency; Combination/Other

Section S3. Client Satisfaction Questionnaire (CSQ-8)

Please help us improve our program by answering some questions about the services you received. We are interested in your honest opinions, whether positive or negative. *Please answer all questions.* Any comments and suggestions are welcome. Thank you very much. We appreciate your help.

Copyright and permission statement: This survey uses items and item responses from the Client Satisfaction Questionnaire© (CSQ-8©, CSQ-3©, CSQ-4©, CSQ-18A/B©), by permission of the copyright holder. Copyright© 2023. Clifford Attkisson, Ph.D. Use, transfer, copying, reproduction, merger, translation, modification, or enhancement (in any version, format, and/or media including electronic), in whole or in part, is forbidden without written permission by Dr. Attkisson. Contact: Info@CSQscales.com.

**CSQ-8 item**	**Response options**
1. How would you rate the quality of the service you received?	Excellent; Good; Fair; Poor
2. Did you receive the kind of services you wanted?	No, definitely not; No, not really; Yes, generally; Yes, definitely
3. To what extent has our program met your needs?	Almost all of my needs have been met; Most of my needs have been met; Only a few of my needs have been met; None of my needs have been met
4. If a friend needed similar help, would you recommend our program to them?	No, definitely not; No, I do not think so; Yes, I think so; Yes, definitely
5. How satisfied are you with the amount of help you received?	Very dissatisfied; Indifferent or somewhat dissatisfied; Quite satisfied; Very satisfied
6. Did the services you received help you deal more effectively with your problems?	Yes, they helped a lot; Yes, they helped somewhat; No, they did not really help; No, they seemed to make things worse for me
7. Overall, in general, how satisfied are you with the services you received?	Very satisfied; Quite satisfied; Indifferent or somewhat dissatisfied; Very dissatisfied
8. If you were to seek help again, would you return to our program?	No, definitely not; No, I do not think so; Yes, I think so; Yes, definitely

Thank you for your time.
